# Ovalbumin oxidative modification fingerprints depend on gas plasma-driven reactive species profiles

**DOI:** 10.1080/13510002.2026.2688623

**Published:** 2026-06-17

**Authors:** Paul Schulan, Kristian Wende, Ramona Clemen, Torsten Gerling, Klaus-Dieter Weltmann, Klaus Ruhnau, Thomas von Woedtke, Michael Lalk, Sander Bekeschus

**Affiliations:** a ZIK Plasmatis, Leibniz Institute for Plasma Science and Technology (INP), A Member of the Leibniz Health Technologies Research Alliance, Greifswald, Germany; b Neoplas MED, Greifswald, Germany; c Institute of Hygiene and Environmental Medicine, Greifswald University Medical Center, Greifswald, Germany; d Department of Biochemistry, Greifswald University, Greifswald, Germany; e Department of Dermatology, Venerology, and Allergology, Rostock University Medical Center, Rostock, Germany

**Keywords:** Hydrogen peroxide, oxidative modification mapping, reactive oxygen and nitrogen species

## Abstract

**Objective:**

Oxidative protein modifications have been linked to several diseases, but the variety and diversity of modifications are less studied.

**Methods:**

We used the chicken egg protein ovalbumin and gas plasma technology, a potent source of various reactive species, for protein oxidation. Using high-resolution mass spectrometry and an in-house workflow, over 80 distinct oxidative protein modifications were mapped at per-amino-acid resolution. To examine how modification profiles depend on changes in reactive species types and concentrations, we generated 12 distinct argon gas plasmas by systematically varying molecular gas admixtures (water, ethanol, oxygen, and nitrogen).

**Results:**

Optical emission spectroscopy (OES) and photometric determination of deposited long-lived species (hydrogen peroxide, nitrite, and nitrate) were applied to profile gas plasma conditions, revealing the admixture-dependent impact on the reactive oxygen/nitrogen species (ROS/RNS) fingerprint. Correlation analysis with mass spectrometry data revealed the significant involvement of atomic oxygen and hydrogen peroxide in protein oxidation. The enrichment of specific reactive species created by a defined gas plasma composition generated specific ovalbumin oxidation profiles resolved per amino acid. Feed gas-dependent oxidation hotspots, such as Trp149 for dry argon gas or Met274 for hydroxyl radical-rich humidified argon gas, were identified.

**Discussion:**

This first-of-its-kind study reveals intricate relationships between dynamic reactive species environments and protein oxidation profiles using ovalbumin as a model system.

## Introduction

Naturally, ROS/RNS are produced endogenously and exogenously and are essential signaling molecules [1,2]. Excessive ROS production or poor antioxidant capacity leads to imbalanced homeostasis. An increasing number of diseases are linked to oxidative stress. Simultaneously, the growing interest in the molecular mechanisms paired with the increasing performance of mass spectrometers and software unveils the role of biomolecule oxidation in these pathologies [3,4]. A potential patho-mechanism is the oxidation of resident proteins close to the site of their formation, leading to immunogenic oxidized antigens [5]. Mimicking pathophysiological conditions is challenging since several and partially very short-lived ROS/RNS are usually involved in oxidative stress processes, e.g. OH• (t_1/2_ = 10^−9^ s) or singlet oxygen (t_1/2_ = 10^−5^ s) [6]. Therefore, it is crucial to have a reliable ROS source capable of producing a wide range of reactive species, ideally without interfering with chemical generation processes. More recently, gas plasma technology was introduced in the field of redox research. By transferring energy into a (carrier) gas, an atmospheric pressure plasma is formed that can be operated at about body temperature [7]. These plasmas are multi-component systems characterized by excited atomic and molecular species, ions, free electrons, UV radiation, thermal radiation, electromagnetic fields, and visible light emission, embedded in a matrix of cold gas atoms or molecules in the ground state. Alongside the established or emerging applications in surface disinfection [8,9], antimicrobial use in dentistry [10,11], and medical applications in wound healing [12,13], this technology has promising applications in redox research as a polypotent and scalable reactive species source. Device settings, such as the applied electrical waveform and frequency, current, or carrier gas composition, allow for modulating the gas plasma chemistry. The ionization energies of the carrier gas, often a noble gas such as argon, helium, or neon, dictate the reactive/excited species created [14]. Additional molecular admixtures can fine-tune the carrier gas, tailoring the reactive species profile [15,16].

Using high-resolution mass spectrometry, we previously provided evidence that gas plasma exposure introduces oxidative modifications (oxMods) into biomolecules, including various proteins, phospholipids, and carbohydrates [17–20]. However, owing to the multiple reaction partners existing within the plasma gas phase, the gas‒liquid interface, and the bulk liquid, a concise match between gas plasma characteristics and associated modification patterns in proteins has not yet been established. To this end, we used the model protein ovalbumin to unravel intricate relationships between 12 different gas plasma reactive species profiles and a set of over 80 (oxidative) modifications resolved at the amino acid level. This setting allowed for performing a correlation analysis between gas plasma characteristics and protein modifications, and this insight can be used to establish targeted oxidation applications when plasmas are applied for redox-related research.

## Materials and methods

### Gas plasma treatment

**Figure 1.**
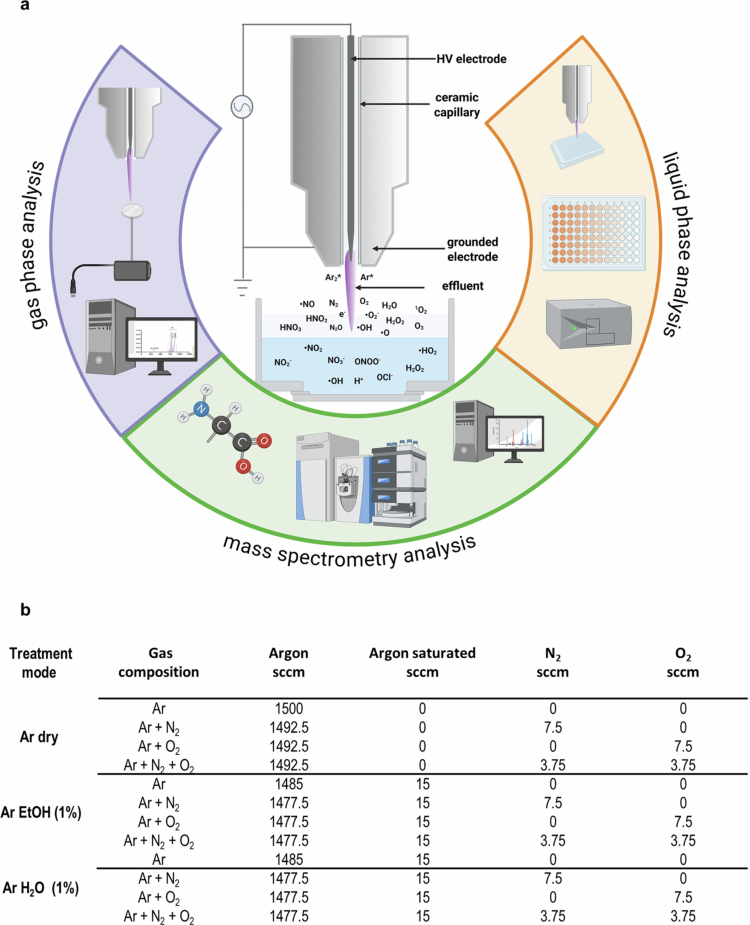
Experimental outline. (a) Scheme of three analytical methods used to evaluate the impact of feed gas composition on gas plasmas and subsequent protein oxidation; (b) iteration matrix of all tested gas compositions given in standard cubic centimeters per minute (sccm), admixtures of water or ethanol were achieved by purging a fraction of the working gas through a respective liquid in a suitable container.

Protein oxidation was performed by gas plasma exposure using the kINPen (Neoplas, Germany). The plasma source working parameters were set to a sinusoidal alternating current (1.1 MHz), a voltage of 2–6 kVp-p, dissipated energy between 1.9–3.2 W, and a gas flow rate of 1.5 standard liters per minute (slm) of argon (Linde, Germany, 2620152, 99.999% purity). Molecular admixtures, such as O_2_ (Linde, Germany, 10100103, 99.999% purity) and N_2_ (Linde, Germany, 2210152, 99.999% purity), were added via an 8-channel high-precision gas flow controller (MKS, Germany). The plasma jet was operated for 30 min before experimental use to ensure reproducible conditions. The tested gas plasma conditions, with the exact gas flows (sscm) shown in Figure 1b, can be categorized into two modes: dry gas mode and humidified gas mode. In humidified mode, 1% of the total working gas flow was saturated with either ethanol (EtOH) (Carl Roth, Germany, K928.2) or double-distilled water (ddH_2_O) by guiding it through a suitable container filled with the respective liquid (bubbler) and added to the main argon stream. At lab temperature, the achieved humidity of ≈280 ppmv closely follows the theoretical absolute humidity expected for a fully saturated carrier gas fraction (313 ppmv at 25 °C), as demonstrated previously [21]. In dry gas mode, the bubbler system was excluded from the gas mixing system. In both modes, additional molecular gas admixtures of N_2_ (0.5%), O_2_ (0.5%), and N_2_ (0.25%) + O_2_ (0.25%) were tested.

### Protein sample preparation

A solution (100 μg/mL) of ovalbumin (chicken) (Lionex, Germany, LET0028) was prepared in 1 × Dulbecco's phosphate-buffered saline (DPBS) (PanBiotech, Germany, P04-36500). Three hundred microliters of protein solution were gas plasma-treated for 20 s in a 96-well flat-bottom plate (Sarstedt, Germany, 83.3924.500). The evaporation effects during treatment were compensated for with ddH_2_O (Table S1). The treatment duration was 20 s in a conductive treatment manner (plasma effluent touching the liquid surface) [22]. The distance between the nozzle and liquid, ranging from 4 to 9 mm, was adjusted for each gas mixture individually to achieve the conductive mode (Table S2). In this mode, the liquid acts as a third electrode, marked by clearly visible and audible changes in the effluent, increased power consumption near the target, and increased deposition of reactive species [23]. While not fully characterized from a physics viewpoint, this mode offers greater reproducibility compared to free or transition modes, and it reflects the current mode in clinical applications. Each gas composition was tested with *n* = 3 biological replicates, each run in three technical replicates. Triplets were pooled and measured in three technical measurements (triple injection). After gas plasma exposure, ovalbumin was reduced with dithiothreitol (30 mM) (Merck, Germany, 1114740005) for 10 min at 90 °C, alkylated with iodoacetamide (60 mM) (Merck, Germany, I1149-5G) for 30 min at room temperature in the dark, and enzymatically digested with sequencing-grade trypsin (Promega, Germany, V5111) at a ratio of 1:20 overnight at 37 °C. Purification and desalting were performed with in-house prepared STAGE-Tips (Fisher Scientific, Germany, 13-110-018). After elution, the samples were concentrated (SpeedVac at 30 °C) and reconstituted in 20 μl buffer A (0.1% formic acid in MS-grade water). Peptide concentration was analyzed (DeNovix, U.S.A., DS-11 FX+), and samples (200 ng/µl) were prepared for mass spectrometry analysis.

### Liquid chromatography-tandem mass spectrometry (LC-MS/MS)

Peptide separation was performed on a nano-LC setup (Dionex UltiMate 3000 RSLCnano) connected to an Exploris 480 mass spectrometer using electrospray ionization (Thermo Fisher, Germany). Peptides were trapped on a PepMap C18 trap column (5 μm particles, 20 × 0.1 mm) (Thermo Fisher, Germany, 164564-CMD) and separated on a PepMap C18 analytical column (3 μm particles, 150 × 0.075 mm) (Thermo Fisher, Germany, 164941) using 0.1% formic acid (Fisher Scientific, Germany, 94318) in MS-grade water (VWR, Germany, 83645.320) (buffer A) and 0.1% formic acid in 95:5 ACN (Merck, Germany, 1.00029.2500): MS-grade water (buffer B). The gradient started at 4% and increased to 40% buffer B over a steady flow rate of 300 nL/min for 50 min, while the column oven temperature was set to 40 °C. The mass spectrometer was equipped with a Nanospray Flex source and a metal emitter tip, which ionized the analytes by electrospray ionization in positive mode (+2 kV). The measurement transfer capillary temperature was set to 250 °C. Data was obtained in DDA mode. The MS1 full-scan parameters were set as follows: 350–1200 m/z, R = 120,000 at 200 m/z, a target of 5 × 10^3^ ions, followed by up to 15 data-dependent MS/MS scans with higher energy collision dissociation (HCD, maximum injection time (IT) 50 ms, isolation width 1.0 m/z, NCE 30%, R = 15,000 at 200 m/z). Dynamic exclusion was enabled and set to 30 s (all Thermo Fisher, Germany).

### Data analysis and protein modeling

DDA-based MS raw data were analyzed using Proteome Discoverer (version 2.4.1.55; Thermo Fisher, Germany). MS/MS spectra were extracted from the raw files, and searches were performed using the FASTA file (ovalbumin: P01012) along with common background contaminants. Oxidative modifications were identified by a plugin to Proteome Discoverer; Byonic (Protein Metrics, U.S.A.), using an in-house list of oxidative modifications based on prior knowledge [24,25] and literature data (Table S3) and the following parameters: MS1 (peptide) mass tolerance = ≤10 ppm, MS2 (fragment) mass tolerance = ≤10 ppm, cleavage specificity = trypsin, missed cleavages = max 2, and maximum number of modifications per peptide = max 2. The false discovery rate was estimated by the software in two dimensions: the protein level and the PSM level (2D-FDR). The identifications were filtered at a 1% cutoff. Since the PSMs were also filtered, a high level of validity of the spectra was ensured. PSMs were further filtered using the following cutoff filters: byonic score ≥ 250 (indicator for correctness of peptide-spectrum match), logProb ≥ 3 (surrogate marker for posterior error probability), and delta mod score ≥ 10 (reflecting confidence in the localization of the PTM within the peptide). A 3D model of ovalbumin was created with Yasara software using the input files from the PDB (Protein Data Bank).

### Optical emission spectroscopy

Optical emission spectroscopy (OES) was performed to map the radiating excited species in the plasma gas phase of the different discharge conditions. The optical fiber was positioned below the effluent in an on-axis position. The distance between the effluent and the entrance lens of the fiber was adjusted for each condition to the same distance as for the liquid treatments (Table S2). The fiber was connected to a spectrometer (Avantes, Netherlands, AvaSpec-3648-USB2) with 0.7 nm spectral resolution (grating 300 lines mm^−2^, slit 10 µm), and spectra were recorded between 200 and 1100 nm. For comparison, the raw spectra intensities were normalized against the most dominant argon peak (764 nm).

### Liquid phase reactive species quantification

Three hundred microliters of DPBS were gas plasma-treated for 20 s in a 96-well flat-bottom plate (Sarstedt, Germany, 83.3924.500). After gas plasma exposure, evaporation was assessed and compensated for with pre-determined amounts of ddH_2_O (Table S1). Hydrogen peroxide (H_2_O_2_) concentrations were quantified with the Amplex Ultra Red assay (Thermo Fisher, Germany, A36006). Fluorescence measurements were executed at λ_ex_ 535 nm and λ_em_ 590 nm using a microplate reader (Infinite F200 pro; Tecan, Switzerland). Nitrite (NO_2_−) and nitrate (NO_3_−) concentrations were assessed with the Griess assay (Biomol, Germany, Cay780001-192). Both assays were performed according to the manufacturer's instructions.

### Statistical analysis

Statistical analysis and graphing were performed using Prism 9.5.1 (GraphPad Software, U.S.A.). Spearman correlation analysis was performed, and *p*-values were adjusted for multiple testing using the Benjamini–Hochberg false discovery rate procedure.

## Results

### Feed gas modified ROS/RNS fingerprints in plasma gas phases

**Figure 2.**
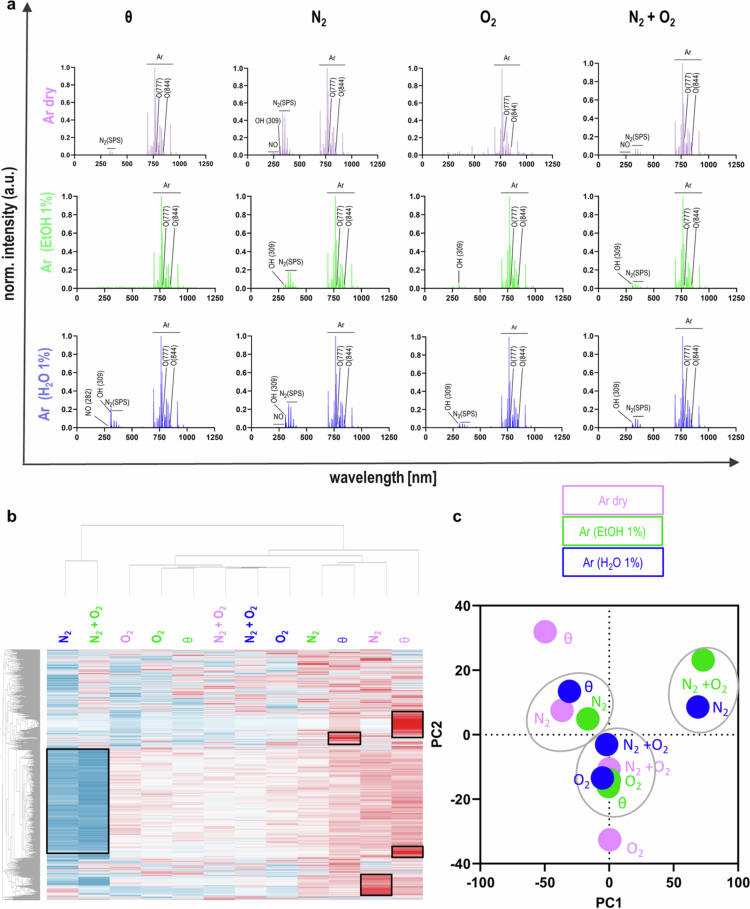
Gas phase optical emission spectroscopy (OES) analysis. (a) OES spectra with highlighted peaks of excited species of interest of several tested gas plasma compositions; (b) hierarchical clustering of OES spectra for each gas composition; (c) principal component analysis (PCA) of emission data of all tested gas compositions.

Since kINPen is a potent multi-ROS source, the fingerprint ROS profiles for each gas plasma composition were determined (Figure 1). Optical emission spectroscopy was used to characterize the excited species in the plasma gas phase (Figure 2a). When operated with each feed gas mode (dry/H_2_O/EtOH) or without any additional admixture to pure-dry argon gas alone (*θ*), all three gas plasma operation modes showed distinct argon peaks that persisted across all subsequent gas compositions. When operated under Ar_dry,_low intensity of the second positive nitrogen system (N_2_ (SPS)) was observed, which was more prominent in Ar_H2O_ and absent in Ar_EtOH_. When molecular N_2_ was added, all three modes showed elevated levels of N_2_ (SPS) peaks, whereas the signals were the highest in the Ar_dry_ mode and the lowest in Ar_EtOH_. Emission lines at 282 nm, reflecting nitric oxide (NO), were observed primarily in an Ar_dry_ + N_2_ discharge. Atomic oxygen lines O (777 nm) and O (844 nm) were observed but remained low in most discharge modes except when O_2_ (0.5%) was added to the feed gas. OH lines (309 nm) were identified in all conditions, with elevated intensities in the humidified modes. When N_2_ and O_2_ were added simultaneously, a combination of both signals was observed. Hierarchical cluster analysis revealed discharge modes with similar emission profiles (Figure 2b). Strikingly, Ar_(H2O)_ + N_2_ and Ar_(EtOH)_ + N_2_ + O_2_ differed the most from all other conditions, while Ar_dry_ + *θ*, as well as Ar_dry_ + N_2_, showed the highest intensities in several wavelength regions. Other discharge modes were placed in between, sharing similarities depending on their gas composition. Within the principal component analysis (PCA), which is composed of the emission intensities of the full spectrum for each condition, the findings were emphasized, showing compositions with similar properties in the clusters, while Ar_dry_ *θ* and Ar_dry_ + O_2_, as well as Ar_(H2O)_ + N_2_ and Ar_(EtOH)_ + N_2_ + O_2_, were separated the most from the group average (Figure 2c). In general, admixtures added to the feed gas in dry mode had the most significant impacts, as all four dry conditions were highly separated along principal component two (PC2), while in humidified conditions, the variation between the different molecular admixtures was less pronounced.

### Feed gas humidification boosted hydrogen peroxide deposition into liquids

**Figure 3.**
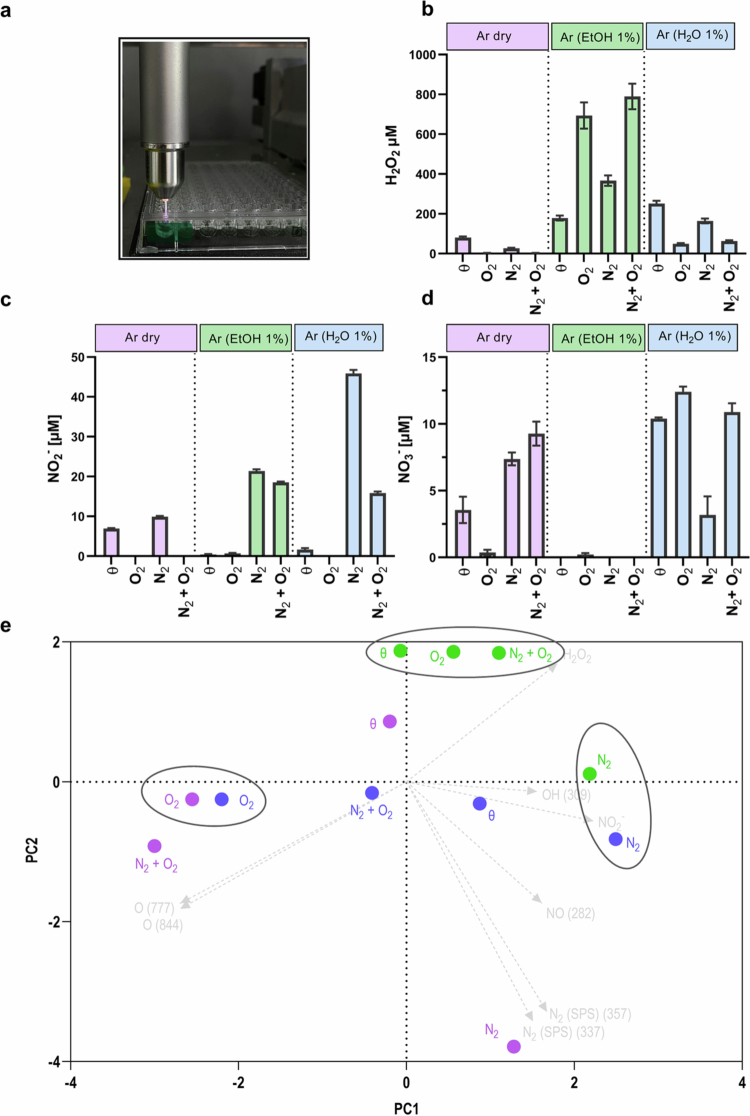
Liquid-phase reactive species assessment. (a) Representative image of gas plasma treatment of PBS in 96-well plate; (b) hydrogen peroxide (H_2_O_2_) concentrations; (c) nitrite (NO_2_^−^) concentrations; (d) nitrate (NO_3_^−^) concentrations; (e) PCA of the three quantified long-lived oxidants for all tested gas compositions.

Gas plasma treatment of liquids was conducted in conducting mode [23], i.e. the plasma effluent was in direct contact with the surface of the treated liquid (Figure 3a). The deposition of H_2_O_2_ showed a substantial variation depending on the plasma gas mode (Figure 3b). For Ar_dry_ + *θ*, a concentration of 60 µM was determined. The deposition increased fourfold when Ar was humidified with H_2_O (≈280 ppmv) and threefold when 1% EtOH-saturated Ar was added to the feed gas. Additional admixture of O_2_ to Ar_H2O_decreased H_2_O_2_ deposition markedly. In contrast, Ar_EtOH_ generated the highest H_2_O_2_ concentrations (750 µM) when molecular gases were added, i.e. N_2_ + O_2_. Comparably high concentrations were produced when only O_2_ was added to Ar_EtOH_. These concentrations were about a log-step higher than those observed in Ar_dry_,. Likewise, the formation of nitrite ions (NO_2_^−^) showed a gas operation mode-dependent production rate (Figure 3c). When neither O_2_ nor N_2_ gas was added, the lowest deposition rate was observed with Ar_EtOH_, while Ar_dry_ yielded the highest rates. The addition of molecular N_2_ resulted in a substantial increase in NO_2_− deposition, with a fourfold increase in Ar_H2O_ + N_2_ and a doubling in Ar_EtOH_ + N_2_ compared to Ar_dry_ + N_2._ Under all conditions, the addition of O_2_ was detrimental. The NO_3_− profile for the three treatment modes revealed comparable concentration when operated in Ar_dry_ mode or Ar_H2O_ but nearly no accumulation of NO_3_^−^ when the feed gas was humidified with 1% EtOH-saturated Ar (Figure 3d). The combined analysis of the liquid analysis data and the OES data unveiled similarities in the ROS profiles for different gas compositions. Notably, three of the four EtOH gas compositions (*θ*, O_2_, N_2_ + O_2_) appeared to be characterized by H_2_O_2_ loadings, clustering at the same level of PC2. In contrast, all other O_2_ admixtures, as well as O_2_ + N_2_, were associated with the atomic oxygen lines present in the OES. N_2_ admixtures in both humidified modes showed a low association with OH^•^ lines in the gas phase and NO_2_^−^ deposition in the liquid, while N_2_ admixture in dry modes showed a strong association with N_2_(SPS) (357 nm; 337 nm) emission (Figure 3e).

### Molecular gas additives altered gas plasma-mediated protein oxidation

**Figure 4.**
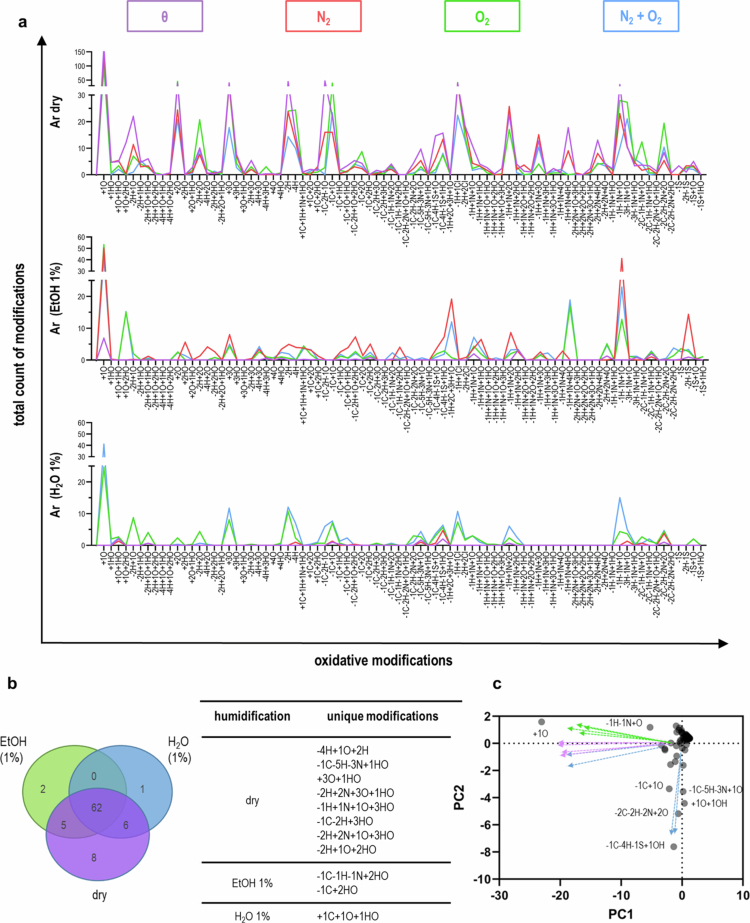
Ovalbumin oxidative modification types and profiles. (a) Ovalbumin oxidative modification counts after gas plasma exposure; (b) Venn diagram exhibiting core oxidative modification set for all gas plasma exposure feed gas modes, the intersection of identical oxidative modifications between treatment modes, and oxidative modifications unique to each gas plasma operation mode; (c) PCA calculated from counts of all identified oxidative modifications for all gas compositions.

In total, 82 different oxidative modifications (oxMods) were identified in OVA across all tested plasma gas compositions, with some occurring more frequently than others. A core of 62 oxMods common to all discharge conditions was determined. In a semi-quantitative approach based on the number of peptide spectra matches (PSMs), the highest number of individual oxMods were found in dry gas plasma modes (up to 150 per oxMod), whereas in H_2_O/EtOH-humidified gas modes, maximum counts were significantly lower (up to 60) (Figure 4a). Alongside, the diversity of modifications was highest in the Ar_dry_ mode and lowest in the Ar_H2O_ mode. In dry argon mode (Ar_dry_), any admixture led to a decrease in most of the detected oxMods (except for −2H + 2O, −4H + 3OH −1C + 2O, −2C − 1H − 1N + 1O, −2C − 2H − 2N + 2O). This was in contrast to ethanol-enriched plasma modes (Ar_EtOH_), where N_2_ or N_2_/O_2_ increased the number of modifications. In humidified argon plasmas, the lowest numbers of modifications were observed for additional admixture of O_2_ or N_2_. Oxidations (+1O), as well as deamidation (−1N − 1H + 1O), occurred commonly in all the treatment modes, but their numbers were reduced in humidified gas plasma modes. Especially in dry gas plasma modes, additional modifications were prominently found, such as dioxidation (+2O) and trioxidation (+3O), didehydrogenation (−2H), loss of formaldehyde (−1C − 2H − 1O), chlorination (−1H + 1Cl), and nitration (−1H + 1N + 2O). If humidified with EtOH, oxidation and hydroxylation (+1O + 2OH), acetylation (−1H + 2C + 3H + 1O), as well as sulfur loss (−1S + 1OH) were the primarily occurring modification types in OVA. In water-humidified argon plasmas (Ar_H2O_), trioxidations (+3O), didehydrogenation (−2H), and chlorination (−1H + 1Cl) were the most frequently introduced modifications besides plain oxidations (+1O) and deamidations (−1H − 1N + 1O), albeit to a lesser extent than in dry argon plasma. Gas plasma with EtOH humidification exhibited two unique oxidation types and shared five additional oxMods with only Ar_dry_. Ar_H2O_ generated one unique oxMod and shares six oxMods with Ar_dry_. Ar_dry_ generated eight unique oxidations detected only in this treatment gas plasma mode (Figure 4b). PCA loading distributions revealed similarities in oxidation profiles for each gas plasma composition and identified two gas compositions (Ar_H2O_ *θ*, + N_2_) that separated from other gas plasmas along the PCA2 axis (Figure 4c).

**Figure 5.**
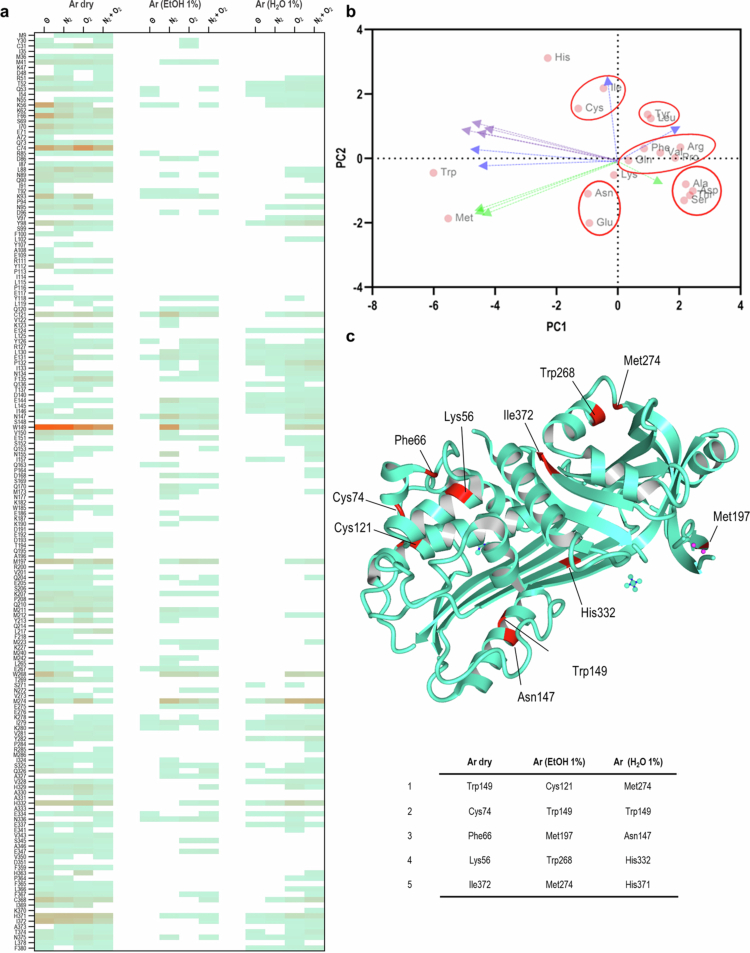
Oxidation profiles at per-amino-acid-resolution. (a) Heatmap of all oxidized amino acid residues displaying how frequently each gas composition oxidized a residue, and with low to high counts represented in green to orange (none: black); (b) PCA of all modified amino acid residues in ovalbumin after gas plasma exposure with loadings for Ar_dry_ colored lila, Ar_EtOH_ in green EtOH, Ar_H2O_ in blue; (c) 3D-model of ovalbumin with the top five oxidized residues for each gas plasma treatment feed gas mode.

To identify OVA modification hot spots, all oxidized residues were visualized in a heatmap (Figure 5a). Ar_dry_ plasma exhibited the strongest oxidation of ovalbumin, with a plethora of modified residues throughout the amino acid sequence. In both humidified modes, a lesser number of modified residues was observed. Of note, the oxidation patterns for EtOH– and H_2_O-enriched plasmas were similar. In dry argon plasma modes, oxidation hotspots were sulfur-containing (Cys74, Met 197, Met274, and Cys368) and aromatic amino acids (F66, W149, W268, His332, and His371). Additional gas admixtures yielded only subtle changes. Humidified gas modes exhibited fewer hotspots of sulfur-containing and aromatic amino acids. This was in line with the results of the oxMods detected for each gas composition, as Ar_EtOH_ generated more oxidized residues if N_2_ was added. In comparison, Ar_H2O_ yielded the highest number of oxidized residues when O_2_ or N_2_ + O_2_ was added. PCA of all modified residues revealed several amino acid clusters. The amino acids scattering most from average were methionine (with a strong Ar_EtOH_ association), tryptophan, and histidine (with Ar_dry_ and Ar_H2O_ associations) (Figure 5b). The locations of the top five oxidized residues on the protein for each treatment are highlighted in a 3D-simulation of ovalbumin. Nearly all oxidations appeared on the protein's surface, facing towards the surrounding liquid (Figure 5c).

### Correlation analysis on gas plasma-derived reactive species and protein oxidation

**Figure 6.**
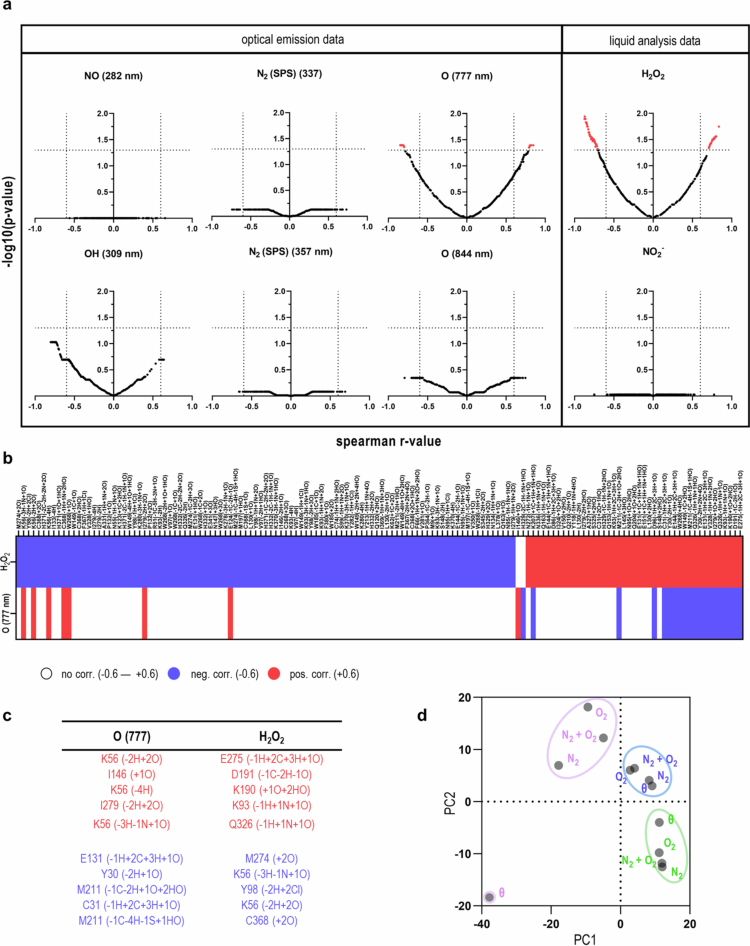
Correlation analysis. (a) Volcano plot displaying the correlation of oxidized protein residues and spectral gas plasma lines from OES, significant (*p* < 0.05) findings with a strong correlation (*r* ≥ 0.6 or *r* ≤ −0.6) highlighted in red; (b) heatmap of significantly positive (red) and negative (blue) correlating oxidized residues; (c) Top five significantly positive (red) and negative (blue) correlating residues with detected oxidative modifications for each reactive species (d) PCA of all data sets obtained in this study.

To potentially enable predictive patterns of gas plasma characteristics and reactive species profiles to drive specific OVA modification fingerprints, correlation analysis was performed between modified residues, including their oxidation type, with reactive species fingerprints of each gas plasma mode. Data points with *p*-values < 0.05 and Spearman *r*-values >0.6 or <−0.6 were considered (red colored dots) (Figure 6a). Atomic oxygen lines (777 nm) exhibited significant findings, which was less the case for both N_2_(SPS) emission lines (337 nm, 357 nm). A large number of negative-correlating data points were identified for residues modified with the OH radical-associated emission line (309 nm); yet, none of them were significant. NO line (282 nm) and deposited NO_2_^−^ showed no significant correlation. In contrast, numerous correlations with H_2_O_2_ deposition were observed. An attempt to assess whether H_2_O_2_ or atomic oxygen (OES line 777 nm) targets specific residues to introduce defined oxidations was made (Figure 6b; red = positive, blue = negative, white = no correlation). The heatmap revealed that H_2_O_2_ and O (777 nm) do not overlap in the oxidation events. Furthermore, the presence of H_2_O_2_ contributed the most to both negative and positive hits. O (777 nm) revealed fewer targets, and demonstrating a contradictory correlation behavior compared to H_2_O_2_ (Figure 6b). The top five modification sites correlated positively or negatively with H_2_O_2_ or O (777 nm) are shown in Figure 6c. Finally, a principal component analysis was generated that included the whole data matrix of each of the twelve plasma feed gas conditions analyzed, as well as protein oxidation and correlation patterns. Again, the dry argon plasma condition was highly distinguished from all the other 11 feed gas conditions (Figure 6d). Strikingly, all dry gas feed gas conditions (except pure argon) clustered together (violet cluster). Similarly, all the water-humidified argon gas conditions built one cluster (blue); so did all the ethanol-humidified gas plasma conditions (green). These findings underlined the rigidity of our approach and data matrix, as differences in plasmas clearly settled in differences in protein oxidation, being dissimilar towards the makeup of the main feed gas (humidified vs. dry), with further molecular admixtures (with oxygen and/or nitrogen) generating more subtle changes.

## Discussion

Redox research in biomedicine has grown increasingly important, since many diseases are linked to oxidative stress [26] and oxidative protein modifications [27,28]. To our knowledge, no system has been described that uses the kINPen plasma device to mimic oxidative stress and to modulate protein oxidation by enriching specific reactive species. This study, therefore, aims to determine whether kINPen is a suitable source of ROS/RNS for addressing redox-related questions by controlling reactive species formation and resultant oxidative protein modifications. Correlating ROS/RNS production with protein oxidation will clarify how molecular admixtures in the feed gas influence oxidative outcomes, ultimately assisting in tailoring gas compositions with predefined oxidation outcomes.

A substantial impact of molecular feed gas admixtures on gas-phase chemistry was observed by optical emission spectroscopy (OES). This rapid and interference-free method allows an initial characterization of the gas phase composition of plasmas, with the limitation that only light-emitting, higher-energy states can be observed. While this is suitable for fingerprinting and discharge quality control, the data do not directly allow quantifications or the conclusion on the absolute gas phase densities and composition. To facilitate the comparison of the different gas plasma discharge modes, the spectra were normalized to the strongest line, according to common procedures, which in the argon-dominated dielectric barrier discharge kINPen is Ar, 774 nm. Other normalization modes were considered, e.g. total spectrum area, but rejected. In the current study, hierarchical clustering of the normalized OES data revealed the formation of gas plasma subgroups with similar gas compositions, which were further substantiated by principal component analysis. The most dominant signals found in all the conditions were argon lines, which is reasonable for an argon-based plasma jet [29]. In the UV region, the most prominent signals belong to the nitrogen second positive system (*C* ^3^Π_u_ → *B* ^3^Π_g_), reflecting the presence of excited N_2_ molecules, especially if N_2_ was added to the feed gas. This shows the presence of a sufficient number of electrons with ≥ 11 eV, weakening the robust triple bond (945 kJ mol^−1^) in molecular nitrogen and increasing the probability of reacting with reactive species, including atomic oxygen and OH•, ultimately yielding reactive nitrogen species [30]. When water was present in the feed gas, a distinctive signal for OH radicals (OH•) appeared at 309 nm. The formation of OH• traces back to several chemical reactions of H_2_O molecules in the plasma, such as dissociative Penning ionization by excited metastable argon intermediates, initially forming H_2_O^+^, which decomposes subsequently, electron-impact dissociation of H_2_O, as well as the participation of H_2_O in the reaction with other ROS species, and UV-induced photolysis of H_2_O to form OH• and other short-lived species [31]. Emission at 777.4 (triplet, 3s ^5^S° → 3p ^5^P) and 844.6 nm (3p^3^ P → 3s^3^ S°) shows the presence of atomic oxygen (O) and correlates with the admixture of O_2_ to the feed gas. Atomic oxygen is formed either by electron-impact dissociation or by Penning ionization with metastable argon species [29]. While water did not suppress O formation, ethanol admix quenched the atomic O signals completely. In this case, a potential competitive reaction is the formation of peroxides from molecular oxygen and alkyl radicals, thereby consuming the available precursor of atomic oxygen. Compared to any feed gas admixture, Ar_dry_ (*θ*) showed the highest emission intensities, i.e. argon lines, which is reasonable since the parameters of the device are fine-tuned to excite argon atoms and no working gas additives interfere with the argon excitation [29].

While OES spectra allow valuable conclusions on the presence of selected primary reactive species and some processes of gas-phase chemistry, subsequent species trajectories cannot be deduced directly. Since an in-depth analysis of gas-phase, interface, or bulk liquid densities of primary and secondary species was out of the scope of this study, the most prominent long-lived species served as proxies for difficult-to-assess short-lived species, e.g. atomic or singlet oxygen, or radical species. Elevated H_2_O_2_ concentrations were detected when the feed gas was enriched with water or ethanol; however, H_2_O_2_-enriching conditions did not correlate with strong or diverse oxPTM patterns in OVA. Despite low OH line intensity in OES, the highest H_2_O_2_ deposition rates were detected for EtOH admixture, suggesting interface or bulk liquid reactions of alkyl radicals or peroxyls to be responsible for H_2_O_2_ formation in this case, as observed before [32]. In this study, elevated H_2_O_2_ levels and potentially highly reactive peracetic acid (PAA) formation occurred when O_2_ was added to an EtOH-humidified gas plasma discharge. The concentrations for the dry and H_2_O-enriched modes are in line with those reported in other studies [33]. Gas-phase H_2_O molecules act as a precursor for the production of H_2_O_2_ by boosting the production of OH•, which then, in turn, can react in a recombination reaction to form the characteristic peroxy bond of H_2_O_2_ [34]. Alongside the high H_2_O_2_ deposition, OES showed strong signals around 309 nm for humidified Ar plasma. The deposition of nitrites and nitrates reflected the availability of suitable precursors, i.e. N_x_O_y_, and the activity of short-lived oxidative species, i.e. OH radicals and atomic oxygen. As a result, a complex pattern with dominant nitrite deposition in humidified argon with N_2_ or N_2_/O_2_ admixture was found. Of note, in the buffered conditions used in this study, the bulk liquid reactions of nitrite, hydrogen peroxide, and protons, yielding peroxynitrite as described for unbuffered aqueous conditions [35], are irrelevant. Instead, the reactions between N_x_O_y_ and O_2_•− at the gas‒liquid interface could lead to its formation [36], and subsequently, to nitration of OVA.

The central objective of the study was to identify and map the oxidative protein modifications induced in OVA by the different plasma discharge conditions. A list containing 80 different oxPTMs was compiled from a literature search, the Unimod website (unimod.org), and previous work [24,25]. To allow identification, a combination of a suitable high-resolution mass spectrometry protocol (data-dependent analysis, DDA) and an advanced search engine designed for post-translational modification detection (Byonic) was employed. To account for rare events, peptide spectral match (PSM) counting was used to quantify the abundance of oxMods, in contrast to precursor ion-based quantification (MS1 level) [37]. While this is a semi-quantitative approach, it is still widely used in proteomics due to the lack of viable alternatives. In contrast to area-under-the-curve-based approaches, which, if a standard compound is available, allow absolute quantification, PSM counts enable the estimation of peptides that have a very low abundance and are only detected in the MS2-mode (fragment ion level). In addition, no standard compound is needed, which, in the case of oxidatively modified peptides, is the most common case. As expected from previous work, the oxMod pattern observed in OVA varied significantly with different discharge conditions, reflecting differences in plasma gas- and liquid-phase chemistry. Under Ar_dry_ conditions, the most dominant oxMods were the addition of one oxygen (+O), loss of two hydrogen atoms (−2H), substitution reactions such as chlorination (+Cl/−H) or nitrations (+NO_2_/−H), and various elimination reactions, e.g. loss of formaldehyde (Figure S1). Ar_H2O_ conditions, with or without admixtures, shared some of the products, but to a much lesser extent. This is in contrast to the significant deposition of H_2_O_2_ or NO_2_^−^ ions in the target liquid, indicating that these long-lived ROS/RNS do not reflect the relevant chemical processes in the liquid. When operated in Ar_EtOH_ + N_2_, an elevated level of acetylation was detected, which was unique to this gas composition. This can be attributed to the presence of peroxyacetic acid, a potential product of ethanol oxidation. The overall trend demonstrated that Ar_dry_ introduced twice as many oxidations compared to H_2_O/EtOH-enriched conditions. Additionally, the variability of the observed oxMods is reduced in these modes.

These findings demonstrate that molecular admixtures to the feed gas modulate and determine the discharge chemistry and, consequently, the type and extent of generated or deposited primary and secondary ROS/RNS in the gas phase and the liquid. Adding molecular gases alters the plasma by reducing the electron density and/or mean electron energy through increased collisional losses. Oxygen is a strong electron acceptor (electronegativity *Χ* = 3.44) and readily forms negative ions (O_2_•−, O^−^). At the same time, nitrogen, despite a similar electronegativity (*Χ* = 3.04), mainly absorbs energy from electrons via elastic collisions (vibrational, rotational, and electronic excitation), forming excited states (N_2_*) instead of negative ions. In both cases, the availability of excited atoms, molecules, or ions promotes downstream chemistry and the formation of additional products [38]. The enrichment of the working gas with H_2_O and EtOH increases the complexity of the plasma discharge due to further modulation of the electron densities and energies and the argon metastable states [39]. Both molecules share the hydroxyl group (─OH) as the reactivity center but differ in the second residue on the oxygen, which is either hydrogen (*Χ* = 2.2) or an alkyl chain (*Χ*_carbon_ = 2.55). The bond energies are 493 kJ mol^−1^ (first O‒H bond in water) or 370 kJ mol^−1^ (O‒C single bond), suggesting that ethanol is cleaved more easily than water, yielding OH• radicals that recombine and form H_2_O_2_, fitting the observed deposition rates. In addition, alkyl or alkoxy radicals are formed, presenting an efficient scavenger for molecular oxygen and oxygen-centered reactive species, yielding peroxides, carboxylic acids, or peracids. This is corroborated by the missing 777.7 nm line in OES and certain oxPTMs in OVA, indicating the limited oxidative capacity of EtOH-enriched conditions. The deposition of nitrite and nitrate ions is generally higher than that in the argon dry mode but shows complex behavior indicative of parallel formation and loss reactions. As an exception, in Ar_EtOH_, no nitrates could be detected, while nitrites were found when molecular N_2_ was present in the working gas. We explain that with the near absence of atomic O and OH radicals, which act as strong oxidants in the gas phase or at the gas‒liquid interface. Despite long-lived species, especially H_2_O_2_, being deposited to a greater extent under humified conditions [16], only a limited correlation with the OVA oxMod pattern was observed. We assume that under humidified conditions, the overall density of ROS besides H_2_O_2_ is low, limiting H_2_O_2_-independent oxidation pathways. Since H_2_O_2_ contributes only to a limited number of oxMods [40], OVA oxidation dropped. One example, where H_2_O_2_ can be one of the main drivers of oxMods, is methionine oxidation to form methionine sulfoxide [41]. Our PCA results indicated that methionine oxidations are highly associated with most of the EtOH gas compositions, a finding in accordance with the high H_2_O_2_ deposition.

The heatmap reveals that a greater variety of amino acid residues are oxidized under dry argon plasma conditions, often to a greater extent. This reflects the activity of short-lived species, which are only represented to a limited extent in our fingerprinting approach. The main indicators are the presence of atomic oxygen lines in the OES. Atomic oxygen reacts with chloride ions in the liquid to hypochlorite, a semi-long-lived strong oxidant (especially under acidic conditions), that yields, among other reactions, chlorination and oxidation of aromatic rings. Alongside, the more prominent presence of nitrate ions in the liquid is an indicator of short-lived species, including NO radicals, at the gas‒liquid interface. Similar to methionine, histidine, and especially tryptophan, are favorable targets for oxidative modifications: under dry conditions, the primary target is Trp149, independent of molecular gas admix. One product, N-formylkynurenine, is evidence for the presence of singlet oxygen [42], which cannot be assessed by OES or robust liquid chemistry [43]. These findings align with previous studies that have identified aromatic amino acids, in addition to sulfur-containing amino acids, as significant targets for oxidation [44–46] The 3D model of the protein highlights the location of the oxMods on the complex protein structure. Nearly all oxMods are located on the solvent accessible surface area (SASA), suggesting a protective role of the protein's 2D/3D structure. Its correct prediction, along with sufficient knowledge of the reaction probabilities of individual amino acid residues with reactive species, could help identify essential targets directly linked to diseases associated with excessive ROS levels in the protein environment and aid in mimicking oxidation scenarios in pathologies.

To further link specific gas compositions to oxMods, a correlation analysis was performed. In contrast to the high levels of deposited NO_2_^−^/NO_3_^−^ as surrogate markers for the presence of RNS, the abundance of identified RNS-induced modifications in OVA is unambiguously low. While a higher N_2_(SPS) signal in the OES can be associated with an increased number of excited N_2_ molecules in the gas phase, this does not necessarily translate into nitrogen species reactivity in the liquid. While weak continuum emission of NO was not observed in OES due to the moderate sensitivity of the setup, NO is one of the first secondary species formed during discharge [36]. Although the molecule is not a substantial contributor to protein oxidation [3], it is a vital reaction partner in forming HNO_2_/NO_2_^−^, which showed a couple of relevant, yet not statistically significant, correlations. For example, NO_2_^−^ in the presence of H_2_O_2_, which can photodissociate into OH•, can lead to the formation of NO_2_• , which preferentially oxidizes tryptophan and tyrosine through a single electron transfer (SET) reaction, attacking the electron-rich aromatic ring [47]. Additionally, NO contributes to the formation of peroxynitrite (ONOO^−^) in the presence of superoxide (O_2_^−^), a strong oxidant and nitrosating agent that participates in protein oxidation. Owing to its short half-life and poor performance of commercial fluorescent kits, which suffer from limited selectivity, it was not determined in this study. Previous work has demonstrated some deposition of ONOO^−^ by kINPen in the target liquid [22,48]. In contrast, significant positive and negative correlations were found between H_2_O_2_ deposition in the liquid, the atomic oxygen lines in OES, and the detection of several oxidative modifications. The oxMods positively correlated with H_2_O_2_ underlined its weak oxidation potential and subsequently limited variation in the mapped oxMods, with polar amino acids (aspartate/asparagine, glutamate/glutamine, and lysine) being the dominant targets. Oxidative deamidation and N- or O-acetylation were the major products observed. The underlying chemistry is either H_2_O_2_ itself (H_2_O-enriched) or reactions involving ethanol oxidation products such as peroxyacetic acid (EtOH-enriched). Conditions inversely correlated with H_2_O_2_ deposition target aromatic (non-polar) amino acids, often tryptophane, histidine, or tyrosine, and indicate that, in conditions where H_2_O_2_ deposition is low (e.g. Ar dry), short-lived ROS such as atomic oxygen or singlet oxygen dominate. Similar trends are seen in an inverted fashion for positive/negative correlations with atomic oxygen lines in OES; however, to a lesser extent, this is due to a lower production in this MHz-driven plasma device. It remains to be noted that singlet oxygen, which is generated in substantial amounts by the kINPen plasma sources in Ar_dry_ mode, was not monitored in the current study [43]. The principal component analysis of all the experiments effectively summarized the significant impacts of the treatment mode and admixtures on the treatment gas. Each treatment mode clusters together in a different quartile of the plot, whereas the gas admixtures fine-tune each gas composition to a unique plasma. This shows that the impact of the used treatment mode (dry vs H_2_O/EtOH enriched) outweighs the effect of the gas admixtures. Ar_dry_, without any admixtures, scatters away from its cluster, as well as from all other gas compositions. This again demonstrates that the gas phase composition significantly impacts the discharge characteristics, gas phase chemistry, and liquid phase chemistry of the formed gas plasma and all subsequent outcomes. In future studies, additional characterization of plasma source parameters, such as electrical properties, can facilitate a better understanding of the interplay between physical, chemical, and biological outcomes driven by feed gas modulations.

With regard to the implementation of gas plasma treatments in medical fields, such as cancer treatments or antimicrobial decontamination, tailoring reactive species generation and deposition in biomedical targets is a central objective in plasma research [29]. Therefore, we compared the impact of twelve different working gas compositions on the protein OVA, which serves as a multi-purpose target. We determined differences in protein modification patterns and found correlations with robust markers of gas-phase and liquid-phase reactive species. Consequently, it can be concluded that the downstream effects of kINPen on a biological target can be controlled by the gas phase composition.

The deposition of H_2_O_2_ scaled strongly with H_2_O or EtOH enrichment of the working gas. This could provide a significant benefit where increased tumor toxicity [49] and a reduced microbial burden are desired. A strong association between methionine oxidation and EtOH-enriched gas compositions was determined, suggesting that this gas composition could be used to model inflammatory conditions in disease research. In the biomedical context, methionine oxidation is associated with ROS stress, leading to the accumulation of methionine sulfoxide (MetO). Higher concentrations of MetO are linked to inflammation- and oxidative stress-related diseases [50]. In addition, acetylation was introduced uniquely for EtOH-saturated feed gas compositions. Protein acetylation plays a vital role in biological signal processes. Pathological alterations are observed in several diseases (e.g. cardiovascular disorders, metabolic syndrome, and cancer), with increased acetylation being linked to disease progression [51]. Plasma can be used to mimic protein acetylation accordingly.

Besides long-lived species, short-lived species have a significant impact on biomedical research. Oxygen-derived radicals such as OH•, atomic oxygen, and superoxide (O_2_−•) are reactive intermediates formed in (patho-) physiological processes, including mitochondrial respiration or myeloperoxidase activity. Hydroxyl radicals are present in different diseases, such as Parkinson's disease [52] or in copper-mediated -OH radical stress in Alzheimer's disease [53]. Although the damaging potential of OH• is well understood, its impact on the development of ROS-stress-associated diseases remains understudied [54]. Their reliable production by chemical processes in biomedical research is notoriously challenging. Selecting a gas composition with high OH• intensities, such as Ar_dry_ + O_2_, could offer a promising source of ROS with reliable production.

ROS are double-edged swords. While high concentrations are associated with oxidative damage to cellular components, such as proteins, lipids, and DNA [55], lower ROS levels mediate stimulating effects [56]. Many ROS-stress-related diseases are linked to oxidized proteins that exhibit loss of function or altered chemical properties, leading to aggregation [57,58]. Some of the detected oxMods in this study are naturally occurring oxidation products under ROS stress and are frequently used as biomarkers of a high ROS burden. For example, 3-nitrotyrosine is commonly found in individuals with high RNS levels, particularly in patients with neurodegenerative diseases (e.g. Alzheimer's disease, Parkinson's disease, Huntington's disease, and prion disease) [59,60]. Similarly, high levels of chlorinated proteins in patient samples indicated elevated myeloperoxidase activity as part of the innate immune response to pathogens [61], yet overshooting MPO activity is linked to chronic inflammation [62]. In addition, peptide backbone cleavages may occur under high-ROS stress but are seldom studied. Highly reactive radicals (e.g. OH•) initiate the abstraction of hydrogen atoms from the α-carbon of the amino acid, subsequently forming carbon radicals that ultimately lead to breaking the backbone of the peptide [63].

The mass spectrometry data revealed feed-gas-dependent protein oxidation, a paramount finding, as the purpose of this study was to verify whether the gas plasma can be tailored to achieve the predefined oxidation outcome. Finally, it is very important to highlight again that kINPen produces a multi-ROS output, making this technology very well-suited to mimic more complex ROS scenarios than a single ROS treatment, which is less biologically relevant, since most ROS stress-related conditions, such as inflammation, are more complex than just a high level of one specific oxidant. (Experimental) manipulation of biomolecules by plasma-driven oxidation can alter their immunogenicity, opening the door to the development and improvement of neoantigen strategies in immunotherapy [64,65].

### Conclusion

To our knowledge, this study is the first to systematically correlate reactive species profiles generated by 12 distinct gas plasma discharge modes with protein oxidation patterns. By integrating OES and liquid-phase assay data with site-specific oxidation analysis, we identified clear oxidation trends that support a more guided selection of gas compositions for inducing directed protein oxidation. This framework may serve as a useful reference for future studies employing gas plasma technology to generate oxidized proteins for functional analyses of oxidative modifications.

## Supplementary Material

Supplementary Material PreScreening_Supplementary_data.docx

## Data Availability

The data that support the findings of this study are available from the corresponding author, S.B., upon reasonable request.
