# Supplementary material for: Ovalbumin oxidative modification fingerprints depend on gas plasma-driven reactive species profiles
Source: Redox Rep. 2026 Jun 17;31(1):2688623. doi: 10.1080/13510002.2026.2688623 (PMC13276820; doi:10.1080/13510002.2026.2688623)
Supplement: Supplementary Material — PreScreening_Supplementary_data.docx [file YRER_A_2688623_SM9095.docx]

**Supplementary data:**

## **Supplementary tables**

## **Table S1**

**Table S1. Evaporation compensation.** Table of added ddH_2_O volumes after gas plasma treatment for each tested gas composition.

|  | added ddH_2_O in µl | | |
| --- | --- | --- | --- |
| gas composition | Ar dry | Ar EtOH | Ar H_2_O |
| Ar | 3 | 6 | 6 |
| Ar + N_2_ | 4 | 7 | 7 |
| Ar + O_2_ | 7 | 9 | 8 |
| Ar + N_2_ +O_2_ | 5 | 8 | 8 |

## **Table S2**

**Table S2. Distances between the nozzle and the liquid used in the gas plasma treatment.** Table of used distances (mm) for each gas composition.

|  | distance of the nozzle to the liquid in mm | | |
| --- | --- | --- | --- |
| gas composition | Ar dry | Ar EtOH | Ar H_2_O |
| Ar | 9 | 6 | 6 |
| Ar + N_2_ | 8 | 6 | 6 |
| Ar + O_2_ | 4 | 5 | 5 |
| Ar + N_2_ +O_2_ | 6 | 5 | 5 |

## **Table S3**

**Table S3. In-house oxidative modification look-up table**. Look-up table with the corresponding mass shift for each potential oxidation on selected amino acids**.**

| **oxidative modification** | **~mass shift (m/z)** | **amino acid** |
| --- | --- | --- |
| +1O | +15.995 | A, D, F, G, H, I, L, P, S, T, V, Y, C, E, K, M, N, Q, W, R |
| +1HO | +17.002 | A, D, F, G, H, I, L, P, S, T, V, Y, C, E, K, M, N, Q, W, R |
| +2O | +31.990 | F, H, I, L, P, S, T, V, Y, C, E, K, M, N, Q, W, R |
| +1O+1HO | +32.998 | F, H, I, L, P, S, T, V, Y, C, E, K, M, N, Q, W, R |
| +2HO | +34.005 | F, H, I, L, P, S, T, V, Y, C, E, K, M, N, Q, W, R |
| +3O | +47.985 | F, H, I, L, P, Y, C, E, K, M, N, Q, W, R |
| +2O+1HO | +48.993 | F, H, I, L, P, Y, C, E, K, M, N, Q, W, R |
| +1O+2HO | +50.000 | F, H, I, L, P, Y, C, E, K, M, N, Q, W, R |
| +3HO | +51.008 | F, H, I, L, P, Y, C, E, K, M, N, Q, W, R |
| -2H+1O | +13.979 | A, D, H, I, L, P, S, T, V, Y, C, E, K, M, N, Q, W, R |
| -2H+1HO | +14.987 | A, D, H, I, L, P, S, T, V, Y, C, E, K, M, N, Q, W, R |
| -1H-1N+1O | +0.984 | Q, N |
| -1H-1N+1HO | +1.992 | Q, N |
| -1H+1N+2O | +44.985 | A, D, F, G, H, I, L, P, S, T, V, Y, C, E, K, M, N, Q, W, R |
| -1H+1N+1O+1HO | +45.993 | A, D, F, G, H, I, L, P, S, T, V, Y, C, E, K, M, N, Q, W, R |
| -1H+1N+2HO | +47.001 | A, D, F, G, H, I, L, P, S, T, V, Y, C, E, K, M, N, Q, W, R |
| -1H+1N+1O | +28.990 | C, K, M, N, P, Q, W, Y, F |
| -1H+1N+1HO | +29.998 | C, K, M, N, P, Q, W, Y, F |
| -1H+1N+3O | +60.980 | F, I, L, Y, W |
| -1H+1N+2O+1HO | +61.988 | F, I, L, Y, W |
| -1H+1N+1O+2HO | +62.996 | F, I, L, Y, W |
| -1H+1N+3HO | +64.003 | F, I, L, Y, W |
| -1H+1N+4O | +76.975 | F, Y, W |
| -1H+1N+3O+1HO | +77.983 | F, Y, W |
| -1H+1N+2O+2HO | +78.991 | F, Y, W |
| -1H+1N+1O+3HO | +79.998 | F, Y, W |
| -1H+1N+4HO | +81.006 | F, Y, W |
| -2H+2O | +29.974 | F, H, I, L, P, R, S, V, Y, C, K, M, Q, W |
| -2H+1O+1HO | +30.982 | F, H, I, L, P, R, S, V, Y, C, K, M, Q, W |
| -2H+2HO | +31.990 | F, H, I, L, P, R, S, V, Y, C, K, M, Q, W |
| -2H+3O | +45.969 | F, I, K, W, Y |
| -2H+2O+1HO | +46.977 | F, I, K, W, Y |
| -2H+1O+2HO | +47.985 | F, I, K, W, Y |
| -2H+3HO | +48.993 | F, I, K, W, Y |
| -1C-1H-1N+2O | +4.979 | H |
| -1C-1H-1N+1O+1HO | +5.987 | H |
| -1C-1H-1N+2HO | +6.995 | H |
| -1H+1Cl | +33.961 | R, N, Q, H, K, F, W, Y |
| -2H | -2.016 | A, D, F, H, I, L, P, S, T, V, Y, C, E, K, M, N, Q, W, R |
| -4H | -4.031 | I, K, L, P, Q, S, T, V, W |
| -1C-2H-1O | -30.011 | D, E, P |
| -1C-5H-3N+1O | -43.053 | R |
| -1C-5H-3N+1HO | -42.046 | R |
| -1S | -31.972 | C |
| -2H-1S | -33.988 | C |
| -1S+1O | -15.977 | C |
| -1S+1HO | -14.969 | C |
| -2C-1H-1N+1O | -23.016 | H |
| -2C-1H-1N+1HO | -22.008 | H |
| -2C-2H-2N+2O | -22.032 | H |
| -2C-2H-2N+1O+1HO | -21.024 | H |
| -2C-2H-2N+2HO | -20.016 | H |
| -1C-2H-2N+2O | -10.032 | H |
| -1C-2H-2N+1O+1HO | -9.024 | H |
| -1C-2H-2N+2HO | -8.016 | H |
| -3H-1N+1O | -1.032 | K |
| -3H-1N+1HO | -0.024 | K |
| +1C+2O | +43.990 | K |
| +1C+1O+1HO | +44.998 | K |
| +1C+2HO | +46.005 | K |
| +1C+1H+1N+1HO | +44.014 | K, C, D, E |
| -1H+2C+3H+1O | +42.011 | K, C, D, E |
| -1C-4H-1S+1O | -32.008 | M |
| -1C-4H-1S+1HO | -31.001 | M |
| -1C-2H+3O | +33.969 | M |
| -1C-2H+2O+1HO | +34.977 | M |
| -1C-2H+1O+2HO | +35.985 | M |
| -1C-2H+3HO | +36.993 | M |
| -1C+1O | +3.995 | W |
| -1C+1HO | +5.003 | W |
| -1C+2O | +19.990 | W |
| -1C+1O+1HO | +20.998 | W |
| -1C+2HO | +22.005 | W |
| -4H+2O | +27.959 | W |
| -4H+1O+1HO | +28.966 | W |
| -4H+2HO | +29.974 | W |
| -4H+3O | +43.953 | W |
| -4H+2O+1HO | +44.961 | W |
| -4H+1O+2HO | +45.969 | W |
| -4H+3HO | +46.977 | W |
| +4O | +63.980 | W |
| +3O+1HO | +64.987 | W |
| +2O+2HO | +65.995 | W |
| +1O+3HO | +67.003 | W |
| +4HO | +68.011 | W |
| -2H+2Cl | +67.922 | W, Y, F |
| -2H+2N+4O | +89.970 | W, Y, F |
| -2H+2N+3O+1HO | +90.978 | W, Y, F |
| -2H+2N+2O+2HO | +91.986 | W, Y, F |
| -2H+2N+1O+3HO | +92.994 | W, Y, F |
| -2H+2N+4HO | +94.001 | W, Y, F |

# Supplementary figures

## Figure S1


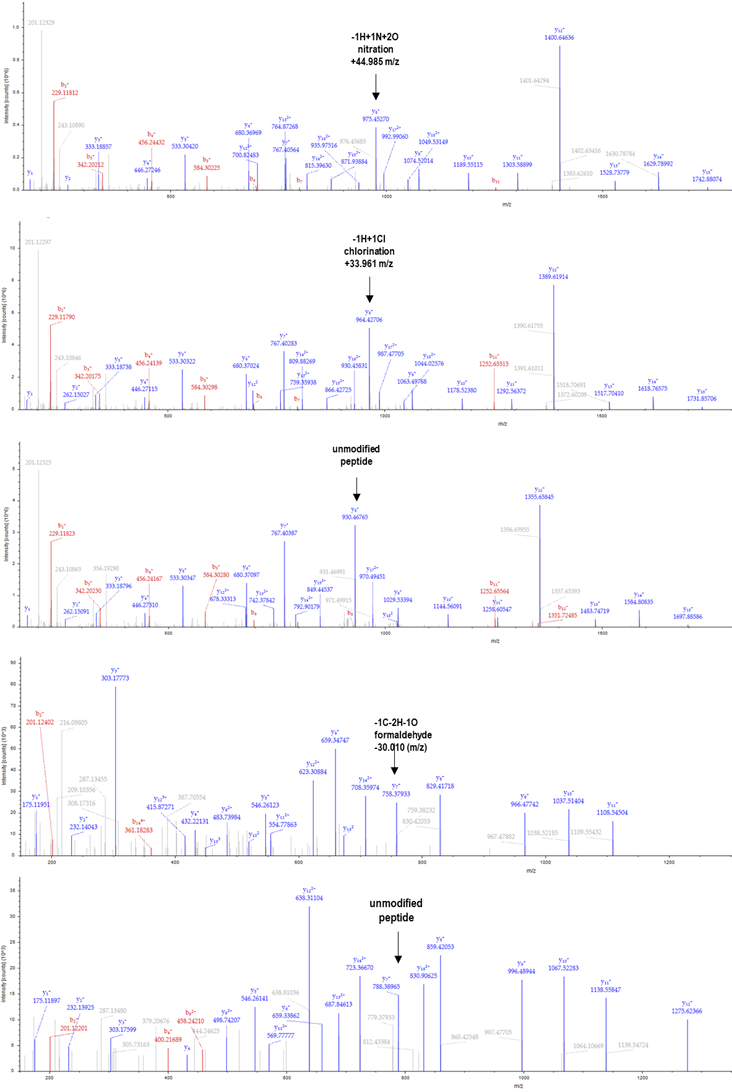


**Figure S1**. **Representative mass spectrometry data.** Representative mass spectrometry data for three different oxMods (nitration, chlorination, loss of formaldehyde) on two different peptides.
